# Plasmodium vivax Invasion of Human Erythrocytes Inhibited by Antibodies Directed against the Duffy Binding Protein

**DOI:** 10.1371/journal.pmed.0040337

**Published:** 2007-12-18

**Authors:** Brian T Grimberg, Rachanee Udomsangpetch, Jia Xainli, Amy McHenry, Tasanee Panichakul, Jetsumon Sattabongkot, Liwang Cui, Moses Bockarie, Chetan Chitnis, John Adams, Peter A Zimmerman, Christopher L King

**Affiliations:** 1 The Center for Global Health and Diseases, Case Western Reserve University, Cleveland, Ohio, United States of America; 2 Department of Pathobiology, Faculty of Science, Mahidol University, Bangkok, Thailand; 3 Department of Biological Sciences, University of Notre Dame, Notre Dame, Indiana, United States of America; 4 Department of Entomology, Armed Forces Research Institute of Medical Sciences, Bangkok, Thailand; 5 Department of Entomology, The Pennsylvania State University, University Park, Pennsylvania, United States of America; 6 Entomology Unit, Papua New Guinea Institute for Medical Research, Papua New Guinea; 7 Malaria Group, International Centre for Genetic Engineering and Biotechnology, New Delhi, India; 8 Global Health Infectious Disease Research Program, Department of Global Health, University of South Florida, Tampa, Florida, United States of America; 9 Veterans Affairs Research Service, Cleveland, Ohio, United States of America; Walter and Eliza Hall Institute of Medical Research, Australia

## Abstract

**Background:**

Plasmodium vivax invasion requires interaction between the human Duffy antigen on the surface of erythrocytes and the P. vivax Duffy binding protein (PvDBP) expressed by the parasite. Given that Duffy-negative individuals are resistant and that Duffy-negative heterozygotes show reduced susceptibility to blood-stage infection, we hypothesized that antibodies directed against region two of P. vivax Duffy binding protein (PvDBPII) would inhibit P. vivax invasion of human erythrocytes.

**Methods and Findings:**

Using a recombinant region two of the P. vivax Duffy binding protein (rPvDBPII), polyclonal antibodies were generated from immunized rabbits and affinity purified from the pooled sera of 14 P. vivax–exposed Papua New Guineans. It was determined by ELISA and by flow cytometry, respectively, that both rabbit and human antibodies inhibited binding of rPvDBPII to the Duffy antigen N-terminal region and to Duffy-positive human erythrocytes. Additionally, using immunofluorescent microscopy, the antibodies were shown to attach to native PvDBP on the apical end of the P. vivax merozoite. In vitro invasion assays, using blood isolates from individuals in the Mae Sot district of Thailand, showed that addition of rabbit anti-PvDBPII Ab or serum (antibodies against, or serum containing antibodies against, region two of the Plasmodium vivax Duffy binding protein) (1:100) reduced the number of parasite invasions by up to 64%, while pooled PvDBPII antisera from P. vivax–exposed people reduced P. vivax invasion by up to 54%.

**Conclusions:**

These results show, for what we believe to be the first time, that both rabbit and human antibodies directed against PvDBPII reduce invasion efficiency of wild P. vivax isolated from infected patients, and suggest that a PvDBP-based vaccine may reduce human blood-stage P. vivax infection.

## Abstract 2

Christopher King and colleagues found that both rabbit and human antibodies inhibited binding of rPvDBPII to the Duffy antigen N-terminal region and to Duffy-positive human erythrocytes, suggesting that a PvDBP-based vaccine may reduce blood stage *Plasmodium vivax* infection.

## Editors' Summary

**Background.:**

Malaria is a parasitic infection transmitted to people through the bite of an infected mosquito. Four different parasites cause malaria—the commonest and most widely distributed of these is Plasmodium vivax. Infections with P. vivax are rarely fatal, but they cause debilitating chills and fevers that recur every other day if untreated. Like other malaria parasites, P. vivax has a complex life cycle. Infected mosquitoes inject a form of the parasite known as sporozoites into people. The sporozoites replicate inside liver cells without causing any symptoms. Then, 8–9 d later, merozoites (another form of the parasite) are released from the liver cells and invade young red blood cells. Here, they replicate rapidly before bursting out and infecting more red blood cells. The characteristic symptoms of malaria are caused by this cyclical increase in the parasite burden. P. vivax infections are usually treated with chloroquine, but patients must also take a second drug called primaquine. This drug kills hypnozoites, a form of the parasite that hibernates in the liver and that can cause a relapse many months after the initial bout of malaria.

**Why Was This Study Done?:**

P. vivax is becoming resistant to chloroquine and, although other antimalarial drugs still kill it, a vaccine that would limit the severity of P. vivax infections by blocking its ability to invade red blood cells is urgently needed. The invasion of red blood cells by P. vivax depends on an interaction between the Duffy antigen (a protein on the surface of human red blood cells) and the Duffy binding protein (PvDBP), which is expressed by merozoites. People who lack the Duffy antigen are resistant to blood-stage infections of P. vivax. Similarly, people who express half the normal amount of Duffy antigen on their red blood cells have reduced susceptibility to these infections. In this study, the researchers investigated whether antibodies (proteins made by the immune system that recognize foreign proteins) directed against PvDBP inhibit the invasion of human red blood cells by P. vivax.

**What Did the Researchers Do and Find?:**

The researchers injected a fragment of PvDBP called PvDBPII into rabbits and purified the part of the blood that contains antibodies from the animals. They also isolated antibodies to PvDBPII from the blood of several Papua New Guineans who had been exposed to P. vivax. Both types of antibodies bound to PvDBPII in test tubes and to PvDBP expressed on P. vivax merozoites. Then, the researchers showed that both types of antibody inhibited the binding of PvDBPII to Duffy antigen when the antigen was in solution and when it was present on human red blood cells. Finally, to test the ability of the antibodies to inhibit red blood cell invasion by *P. vivax,* the researchers established short-term cultures of the parasite from blood taken from infected adults living in Thailand. Addition of the rabbit or human antibodies to these cultures inhibited parasite invasion of red blood cells by more than 50%.

**What Do These Findings Mean?:**

These findings show, for what is believed to be the first time, that antibodies recognizing a fragment of PvDBP can partly inhibit the invasion of red blood cells by P. vivax merozoites. The results with the human antibodies are particularly important as they strongly suggest that a PvDBP-based vaccine might provide protection against blood-stage P*. vivax* infections. Whether the level of inhibition of invasion seen in this study will be sufficient to reduce the clinical severity of these infections will only become clear, however, when a vaccine is tested in people. The findings also indicate that short-term P. vivax cultures can be used to test whether antibodies that recognize other merozoite proteins also inhibit invasion. Unlike P. falciparum (the other major malarial parasite), P. vivax cannot be grown continuously in the laboratory. These short-term cultures will at last provide vaccine developers with a way to evaluate antigens as candidates for inclusion in *P. vivax* vaccines.

**Additional Information.:**

Please access these Web sites via the online version of this summary at http://dx.doi.org/10.1371/journal.pmed.0040337.

- MedlinePlus encyclopedia page on malaria (in English and Spanish)
- Information from the US Centers for Disease Control and Prevention on malaria (in English and Spanish)
- Vivaxmalaria, information for the malaria research community on topics related to Plasmodium *vivax*
- Information from the Malaria Vaccine Initiative about malaria and malaria vaccines, including a fact sheet on *Plasmodium vivax* malaria

## Introduction

Plasmodium vivax accounts for at least half of all malaria cases in Latin America, Oceania, and Asia [1]; 70 to 80 million clinical P. vivax cases occur worldwide annually. While Plasmodium falciparum uses a complex array of receptors to invade human erythrocytes [2–6], erythrocyte invasion by *P. vivax,* and the closely related simian parasite *Plasmodium knowlesi,* are understood to depend upon interaction with the Duffy blood group antigen [7,8]. In the homologous P. knowlesi system, merozoites interact with Duffy-negative human red blood cells, but are unable to invade [8,9]. In Africa, where Duffy-negativity has reached fixation in many different ethnicities, transmission of P. vivax malaria is uncommon [1,10]. Of further interest, in Papua New Guinea, heterozygous carriers of a Duffy-negative allele are shown to express half the amount of the Duffy antigen on erythrocytes compared to wild-type homozygotes [11], and exhibit reduced susceptibility to P. vivax blood-stage infection [12]. These observations suggest that completely or partially disrupting access to the Duffy antigen reduces the ability of the parasite to invade new erythrocytes and may constrain P. vivax parasitemia.

The Duffy antigen shares structural features with chemokine receptors (which have the alternative name of Duffy antigens/receptors for chemokines [DARC]) [13], and exhibits binding to a unique array of chemokines [14–16]; however, because the Duffy protein has no known signaling function, it is no longer included in the chemokine receptor nomenclature system [17]. The Duffy binding protein (DBP), a 140-kD transmembrane protein, serves as the parasite ligand in P. vivax and P. knowlesi erythrocyte-invasion complexes [18–21]. The protein is characterized by two cysteine-rich regions (II and IV) sharing amino acid sequence homology with other malaria parasite erythrocyte-binding ligands [2]. To date, P. vivax DBPII and orthologous P. knowlesi DBPα (71% sequence identity) are the only parasite ligands known to bind Duffy [20,21]. A number of competitive binding [16,22] and gene-deletion [23] strategies have demonstrated the importance of the DBP–Duffy interaction in regard to a range of parasite–host processes leading to erythrocyte invasion. Of greatest relevance to our current study, antibodies generated against P. knowlesi DBPα inhibit P. knowlesi invasion of both human and rhesus erythrocytes in vitro [24]. At present, it is not known whether antibodies against region two of the P. vivax Duffy binding protein (PvDBPII) can also inhibit erythrocyte invasion of P. vivax.

Recent advances have made it possible to express refolded recombinant region two of the P. vivax Duffy binding protein (rPvDBPII), which exhibits the Duffy antigen-binding characteristics of the full-length parasite protein [25]. With this purified protein, it has become possible to develop PvDBPII-specific antibodies for further studies evaluating P. vivax Duffy binding protein (PvDBP) [26]. Additionally, recent progress in culturing P. vivax field isolates in vitro [27,28] presents new opportunities to improve understanding of the mechanisms of P. vivax invasion and the ability of antibodies directed against merozoite antigens to inhibit parasite invasion and/or growth in erythrocytes. More specifically, we aim to determine whether molecular inhibition of PvDBP–Duffy binding translates into inhibition of P. vivax invasion of human erythrocytes. Here, we conducted a series of in vitro studies to purify rabbit and human PvDBPII-specific polyclonal antibodies that inhibit PvDBP–Duffy binding. We then used these reagents to test the hypothesis that human PvDBPII-specific antibodies are able to inhibit in vitro invasion of human erythrocytes by *P. vivax.*

## Materials and Methods

### Human Blood Samples

All human blood samples used in this study were collected after obtaining consent from study participants under protocols approved by the Ethical Review Board of the Cleveland Veteran's Administration Medical Center, the Papua New Guinea Medical Research Advisory Committee, the Ethical Review Committee of Mahidol University, the Thai Ministry of Public Health, and the United States Army.

For short-term culture studies, blood infected with P. vivax was collected from adult males from an endemic area along the Thailand/Myanmar border who presented with clinical malaria at the Mae Sot clinic. P. vivax infection was confirmed by thick- and thin-smear blood films using standard Giemsa staining techniques and light microscopy [29,30]. Coinfection by other malaria species was ruled out by light microscopy and OptiMAL antigen-capture stick tests [31]. Plasma samples for isolating human anti-PvDBPII Ab (antibodies against region two of the Plasmodium vivax Duffy binding protein) were obtained from P. vivax–exposed patients (aged 12–43 y) from the Wosera region of the East Sepik province, a malaria-holoendemic area of Papua New Guinea [32]. All blood was collected in heparin or EDTA vacutainers and used as whole blood for parasite culture or as plasma to isolate human anti-PvDBPII Ab (cryopreserved at −80 °C).

### Expression, Refolding, and Purification of Recombinant PvDBPII

Production and purification of recombinant P. vivax DBPII variants (Salvador 1 [Sal 1] and C [33]) followed methods described previously by Singh et al*.* [25]. Details of the experimental approach used and the results obtained are elaborated in Figure S1. Recombinant PvMSP1_19_ (Plasmodium vivax merozoite surface protein-1 19) was kindly provided by T. Stowers of the Malaria Vaccine Unit, National Institute of Allergy and Infectious Diseases, at the US National Institutes of Health.

### Preparation of Anti-PvDBPII Ab

Recombinant PvDBPII (100 μg) was injected into rabbits intramuscularly at 3-wk intervals, emulsified in Titermax Gold (CytRx). The IgG fraction of the serum was purified using a protein-G column. Cryopreserved plasma samples from adults residing in P. vivax–endemic areas of Papua New Guinea were initially screened for the presence of anti-PvDBPII Ab (below). Plasma samples were pooled from P. vivax–exposed individuals (*n* = 14; with ELISA optical density values five times greater than in unexposed individuals), and from P. vivax–unexposed individuals (*n* = 7). Human anti-PvDBPII Ab was affinity purified by passing the clarified pooled human plasma over an affinity column made by binding 5 mg of the rPvDBPII protein to cyanogen bromide–activated sepharose beads. The column was washed with three volumes of PBS, pH 7.4, and bound antibodies were released from the column using an elution buffer (0.1 M glycine-HCl, pH 3.5); the antibody-containing solution was immediately neutralized by adding 0.1 volumes of 1 M Tris-HCl (pH 8.5) and then dialyzed against PBS. The amount of total IgG in the eluate and its ability to bind to the rPvDBPII were determined by ELISA.

### ELISA-Based Binding-Inhibition Assay

A construct encoding the N-terminal 60 codons of the human DARC protein was ligated to the sequence encoding the Fc region of human IgG (nDARC-Ig) and cloned into the mammalian expression vector pCDM8 [34–36]. As Choe et al*.* have demonstrated that sulfonation of tyrosine at amino acid 41 of the human Duffy antigen is essential for interaction with rPvDBPII, nDARC-Ig was expressed following cotransfection of mammalian cells with plasmids encoding nDARC-Ig and human tyrosyl protein sulfotransferase-2 [34]. The expressed recombinant protein was purified from cell culture supernatants by affinity chromatography using Protein A (Pierce). The nDARC-Ig chimeras were further purified by gel filtration chromatography using Superdex 200 (Amersham Biosciences) in PBS together with 300 mM NaCl. Recombinant nDARC-Ig (1 μg/ml) in 50 μl of NaHCO_3_ (pH 9.6) was added to Immulon 4 ELISA plates and incubated overnight at 4 °C. Recombinant PvDBPII protein (0.1 μg/ml) was added to allow binding to nDARC-Ig for 2 h at 37 °C. Bound rPvDBPII was detected with rabbit anti-PvDBPII serum (serum containing antibodies against region two of the Plasmodium vivax Duffy binding protein) (1:8,000 dilution) followed by an alkaline phosphatase–conjugated goat anti-rabbit antibody (1:5,000 dilution; Jackson ImmunoResearch). Binding-inhibition experiments were performed by pre-incubating rPvDBPII (0.1 μg/ml) with rabbit or human antibodies for 1 h at 37 °C before adding to the nDARC-Ig–coated plate. The mAb Fy6 (50 μg/ml), recognizing N-terminal amino acids 19–25 of the Duffy antigen [37,38] inhibited binding of rPvDBPII to human erythrocytes as expected at levels comparable to those observed for the anti-PvDBPII Ab (unpublished data). All parallel experiments were run with Duffy-negative cells and detectable binding was always <5% (unpublished data). Fy6 [39] was obtained from BD Biosciences Pharmingen.

### Erythrocyte-Binding Assays

Assays were performed by incubating donor erythrocytes (10^6^) with rPvDBPII (1 μg) for 4 h at room temperature or overnight at 4 °C in 100 μl of PBS together with 1% BSA. Each sample was washed three times with PBS and 1% BSA and incubated (1 h in the dark at 4 °C) with mouse anti-HIS antibody (1:25 dilution) conjugated to Alexa Fluor 488 (Qiagen). Samples were then washed four times with PBS plus 1% BSA and resuspended in the same solution (200 μl). LSRII-based (Becton-Dickinson) flow cytometry evaluated 50,000 cells. To evaluate the ligand–receptor inhibitory ability, rabbit and human anti-PvDBPII Ab were incubated with rPvDBPII at the specified dilutions for 1 h at 37 °C before combining with donor erythrocytes. Percent binding was evaluated by assessing the percentage of erythrocytes with bound rPvDBPII following exposure to test serum divided by the percentage of erythrocytes with bound rPvDBPII following exposure to pre-bleed serum (rabbit) or equivalent concentrations of purified human IgG from non-malaria exposed individuals and multiplied by 100.

### Immunofluorescent Microscopy

Blood from patients infected with P. vivax or P. falciparum parasites was added to a 60% Percoll column and centrifuged at 1,000*g* for 10 min [28,40] to enrich collection of erythrocytes infected with viable schizont-infected erythrocytes. For immunofluorescent microscopy, thin-smear preparations were made on glass slides and fixed with cold acetone for 10 min at −20 °C. After drying, slides were blocked with reconstituted 5% w/v powdered milk for 10 min at 37 °C in a moist dark environment. Slides were then washed twice with PBS. The primary anti-PvDBPII Ab was then applied and incubated for 15 min as described above. After washing twice with PBS, goat anti-rabbit conjugated to FITC (1:1,000) and Hoechst 33342 (0.01mg/ml; DNA stain) were added. Slides were incubated for 30 min, then washed three times in PBS and allowed to dry in the dark. Fluorescent stains were fixed onto the slides using Slow-Fade anti-fade (Molecular Probes) and visualized using an Olympus 100× oil-immersion lens.

### P. vivax Invasion-Inhibition Assay

In vitro invasion assays were performed by first collecting ∼5 ml of whole peripheral venous blood (heparin vacutainer) from two adult P. vivax–infected donors visiting the Malaria Clinic in Mae Sot, Thailand. Donated blood was washed three times in McCoy's 5A medium (Sigma) supplemented with 25 mM HEPES, 0.25% NaHCO_3_, 2.2 mM l-glutamine, 0.08 mg/ml gentamicin, and 25% human AB serum (PvCM and AB25). After washing, the blood was depleted of leukocytes using a CF 11 cellulose powder (Whattman) column [27], resuspended in PvCM plus AB25 at a 5% hematocrit, and transported to the laboratory in Bangkok at ambient temperature within 48 h. P. vivax–infected cultures (10 ml) were grown at 37 °C in 5% CO_2_, 5% O_2_, and 90% N_2_ and were maintained by replacing the PvCM plus AB25 daily until predominately late-stage P. vivax–infected erythrocytes were observed. At this point, the parasite cultures were divided and added to individual wells of 96-well microtiter plates (200 μl), at 5% hematocrit, to evaluate invasion in the presence or absence of antibodies. To maximize the amounts of antibodies that were tested in Experiment 1, human AB serum in the culture was lowered to 20% (PvCM and AB20). Rabbit anti-PvDBPII serum, diluted 1:10, or human anti-PvDBPII Ab diluted to concentrations of 37 and 150 μg/ml was added upon initiation of the P. vivax cultures using the erythrocytes from the first patient. Experiment 2 utilized the standard amount of human AB serum (25%), culturing the parasites in PvCM plus AB25 with the erythrocytes from the second patient and diluting rabbit anti-PvDBPII serum 1:100, or 1:1,000. Experiment 2 utilized human anti-PvDBPII Ab, which was diluted to the final concentrations of 25 and 100 μg/ml. All cultures were grown at 5% hematocrit as described above for 24 h. To assess the number of new P. vivax invasion events, multiple thin smears were prepared from each culture condition (triplicate wells), and then fixed in 95% ethanol before staining with 4% Giemsa (Sigma). To determine parasitemia, a total of 200 high-powered microscope (100× oil immersion) fields were counted with approximately 100 erythrocytes per field for each condition (∼20,000 red blood cells). The microscopist was blinded to the experimental conditions. All infected erythrocytes were counted and classified from early ring forms through schizont and gametocyte stages. For these parasite cultures initiated with predominantly schizont developmental stages, any rings through early trophozoites observed after culturing were considered to be new invasions since these developmental stages are known to occur within the 24-h time frame of the short-term culture.

### Statistical Analysis

Independent two-sided Student's *t*-tests for equal variances were performed using GraphPad Prism version 4.0 (GraphPad Software) to assess differences in binding inhibition between mean values of control and experimental treatments. *p*-Values of less than 0.05 were considered significant.

## Results

### Anti-PvDBPII Ab Inhibit Binding of rPvDBPII to Duffy Antigen In Vitro

Our studies were initiated by expression of rPvDBPII containing the minimal binding region for parasite binding to the Duffy antigen. The antibodies specific for this protein were characterized using serial dilutions to determine end-point titers for both rabbit and human anti-bodies directed to PvDBPII (Figure S2). Preferential enrichment for human antibodies to rPvDBPII by affinity purification was confirmed by marked reduction in antibodies directed against PvMSP1_19_ (Figure S2B) and failure of enriched antibodies to recognize fixed P. falciparum trophozoites and schizonts by immunofluorescence microscopy (unpublished data).

**Figure 1:**
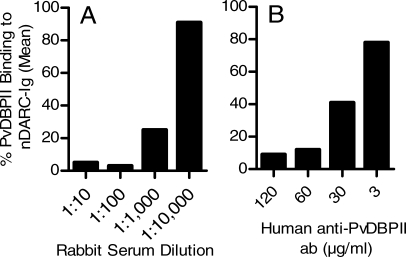
Inhibition of nDARC-Ig Binding to rPvDBPII by Antibodies Rabbit (A) and human (B) anti-PvDBPII Ab were tested to observe inhibition of the interaction of the rPvDBPII protein and the N-terminal region of Duffy in the nDARC-Ig chimera. In ELISA-based nDARC-Ig assays, bars indicate the mean binding percentage relative to pre-bleed rabbit serum or nonspecific human IgG (120 μg/ml). Duplicate experiments showed variation of <5%.

As interpretation of the protein–protein interactions involved in ligand–receptor binding and further antibody-based interference has the potential to be complex, we analyzed PvDBPII–Duffy binding by three different in vitro assays. As a first approach, we evaluated rPvDBPII–Duffy interaction in a cell-free system initially described by Choe et al*.* [34]. Here, the rPvDBPII was allowed to interact with the 60 N-terminal amino acids of the Duffy antigen of the chimeric protein, nDARC-Ig. The results presented in Figure 1A and 1B show that the rabbit polyclonal and affinity-purified human anti-PvDBPII Ab each inhibit rPvDBPII binding to nDARC-Ig in a dose-dependent fashion similar to that observed in previous binding assays. Levels of rabbit and human antibodies corresponding to 50% inhibition of binding were less than the 1:1,000 dilution rate and were between 3 and 30 μg/ml, respectively.

As a second approach, we evaluated PvDBPII–Duffy binding and antibody interference by observing interaction between PvDBPII on the surface of transfected COS7 cells [41] and erythrocytes from Duffy-positive donors. Binding of the erythrocytes to the COS7 cells was assessed by the formation of rosettes. The disruption of the PvDBPII–Duffy interaction was indicated by the absence of rosette formation. Pre-incubation of COS7 PvDBPII transfectants with a 1:3,200 dilution of the rabbit anti-PvDBPII serum inhibited binding to erythrocytes from Duffy-positive donors by 50% compared to the pre-bleed serum (see Figure S3).

**Figure 2:**
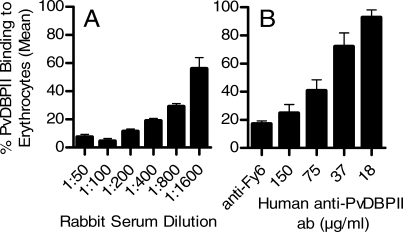
Inhibition of rPvDBPII Binding to Human Red Blood Cells by Antibodies Rabbit (A) and human (B) anti-PvDBPII Ab were tested to observe inhibition of the interaction of the rPvDBPII protein and Duffy-positive human erythrocytes. In erythrocyte-binding assays, the bars indicate the mean binding percentage of four separate experiments (± standard deviation) relative to pre-bleed rabbit serum or nonspecific human IgG (150 μg/ml). In parallel experiments run with Duffy-negative cells, binding was always <5% (unpublished data). Fy6 antibodies (50 μg/ml; recognize N-terminal region of the Duffy receptor) were used to show relative inhibition compared to the affinity-purified human anti-PvDBPII Ab.

Finally, we use a flow-cytometric approach to assess binding of rPvDBPII to Duffy-positive erythrocytes and binding inhibition by both rabbit and human anti-PvDBPII Ab. For this assay, refolded rPvDBPII protein was incubated with test serum prior to mixing with Duffy-positive or -negative erythrocytes. Increasing the dilutions of rabbit anti-PvDBPII serum inhibited binding to erythrocytes relative to pre-bleed rabbit serum in a dose-dependent fashion (Figure 2A). Addition of varying concentrations of human IgG enriched for anti-PvDBPII Ab also inhibited binding in a dose-dependent fashion (Figure 2B). Similar to the nDARC-Ig ELISA results described above, we observed that 50% binding inhibition was observed for rabbit and human antibodies, corresponding approximately to a 1:1,000 dilution and to a range of 37–75 μg/ml, respectively.

### Antibodies Recognize Native PvDBPII of P. vivax Merozoites

**Figure 3:**
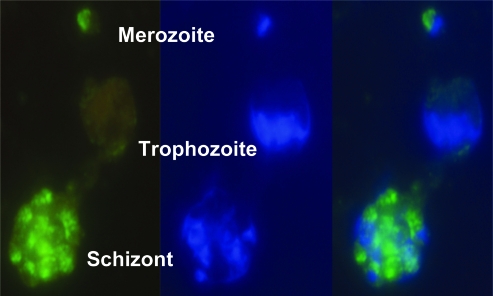
Rabbit Anti-PvDBPII Ab Staining of P. vivax Merozoites in Infected Human Erythrocytes Rabbit anti-PvDBPII serum binding to fixed P. vivax merozoite, trophozoite, and schizont (uninfected cells not shown) was confirmed by immunofluorescence microscopy. Parasites were enriched by Percoll gradient centrifugation from a Thai patient infected with P. vivax. In the left panel, PvDBPII is stained green by a 1:10 dilution of rabbit anti-PvDBPII serum, followed by FITC-conjugated goat anti-rabbit antibody. In the middle panel, DNA is stained blue by Hoechst. The right panel shows the overlay of the left and middle micrographs. Additionally, the antibody concentrated anterior to the nucleus towards the apical end of the merozoite where PvDBPII is known to be expressed in the micronemes [18].

We were interested to determine whether the affinity-purified anti-PvDBPII Ab reacted with native PvDBP of merozoites. DBP is sequestered in the microneme apical organelles until invasion is initiated [18]. Therefore, we expected to see immunofluorescence localization anterior to the nucleus in mature merozoites of late-stage schizonts. To evaluate anti-PvDBPII Ab recognition of native parasite PvDBP, we applied rabbit anti-PvDBPII serum to thin-smear preparations of schizont-enriched P. vivax samples. We also incubated these same preparations with the DNA-specific dye, Hoechst 33342, to identify nuclei of individual merozoites. The immunofluorescence microscopy results presented in Figure 3 illustrate that the rabbit anti-PvDBPII serum binds to the apical end of the P. vivax merozoite where PvDBP expression would be expected to occur [18]. In this same preparation, our immunofluorescence results also show anti-PvDBPII Ab recognition of developing merozoites within a schizont-infected erythrocyte as well as the absence of antibody recognition of structures within trophozoites. Similar results were observed with the human anti-PvDBPII Ab affinity purified from P. vivax–exposed individuals from Papua New Guinea (unpublished data).

### Anti-PvDBPII Ab Decrease P. vivax Human Erythrocyte Invasion

Finally, with results suggesting that the human and rabbit anti-PvDBPII Ab–specific sera recognize native PvDBP and inhibit its function, we wanted to test whether these antibodies inhibit P. vivax invasion of human red blood cells. For these studies, we evaluated the progress of P. vivax development from the late schizont stage for 24 h to enumerate the appearance of ring and trophozoite stages that would signal new red cell–invasion events. Table 1 shows the results of two successful parasite invasion trials, in which parasites were cultured in the absence of the anti-PvDBPII Ab for 24 h. Over this time period, the percentage of schizont and late-trophozoite developmental stages decreased as the parasites matured and ruptured their host cells, while the number of rings, early trophozoites, increased resulting in a doubling in parasitemia—all of which suggest successful invasion of new red blood cells. These results provided a point of comparison for evaluating the inhibition by anti-PvDBPII Ab of red cell invasion by P. vivax merozoites.

**Table 1:**
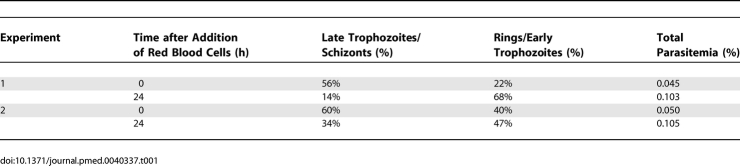
Invasion of Human Red Blood Cells by P. vivax In Vitro

**Figure 4:**
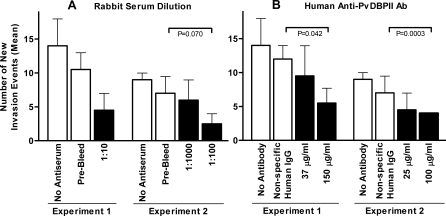
Inhibition of P. vivax Invasion of Human Red Blood Cells by Anti-PvDBPII Ab Tests were performed to examine the influence of rabbit (A) and human (B) anti-PvDBPII Ab on P. vivax invasion on a patient sample cultured in PvCM plus 20% AB serum (Experiment 1) or a sample from a second patient cultured in PvCM plus 25% AB serum (Experiment 2). Control cultures (white bars) contained media without antibodies and were the same for both experiments. The concentration of antibodies (pre-bleed rabbit serum or nonspecific human IgG) in the positive control cultures (white bars) was equal to the highest concentration of test serum. Various concentrations of the test antibody (black bars) were added to late-stage P. vivax cultures and grown for 24 h (in duplicate), and the number of newly invaded cells was observed by light microscopy based on examination of 200 high-powered fields of Giemsa-stained thin smears or approximately 20,000 erythrocytes. Bars indicate mean ± standard deviation of the number of invasion events. *p*-Values (two-sided *t*-test) are shown for differences of *p* < 0.1 between test samples and their respective control antibody.

For the P. vivax invasion-inhibition experiments, we exposed schizont-enriched infected blood to the rabbit and human affinity-purified anti-PvDBPII Ab on two separate occasions and counted infected cells. The results presented in Figure 4 show a reduction of newly invaded red blood cells of up to 64% (1:100) by the rabbit anti-PvDBPII serum relative to pre-bleed serum (Student's *t*-test, *p* = 0.070). When the affinity-purified human anti-PvDBPII Ab preparation was added to these short-term in vitro cultures, we observed a reduction in new P. vivax invasion events by 47% (100 μg/ml) and 54% (150 μg/ml) when compared to cultures exposed to nonspecific human IgG obtained from individuals from non-endemic areas (Student's *t*-test, *p* < 0.001 and *p* = 0.042, respectively). In both of the experiments, rabbit and human anti-PvDBPII Ab demonstrated a dose-dependent inhibition of P. vivax merozoite invasion of human red blood cells. The antibody level inhibiting P. vivax invasion events for the rabbit anti-PvDBPII serum was between the 1:10 and 1:100 dilutions, and for the human anti-PvDBPII Ab the level was between 100 and 150 μg/ml.

## Discussion

This study demonstrates that anti-PvDBPII Ab obtained from humans exposed to *P. vivax,* or artificially induced in rabbits, can partially inhibit P. vivax merozoite invasion in short-term cultures. Our results establish region II of PvDBP as a prominent ligand engaging the Duffy antigen on human red blood cells, making it a potential vaccine candidate against P. vivax. These studies also demonstrate, for what we believe to be the first time, the utility of short-term P. vivax cultures derived from human isolates for measuring the invasion-inhibitory potential of antibodies directed against a specific merozoite antigen. This approach can be used to test additional antibodies targeting other P. vivax merozoite invasion ligands to evaluate potential alternative antigens as vaccine candidates similar to studies performed more routinely for P. falciparum [42,43].

In addition to these results, the same anti-PvDBPII Ab were used in three different binding-inhibition experiments. Comparing results obtained using these strategies provided the opportunity to examine potentially complex PvDBP–Duffy antigen interactions through cell-free and cell-based assay systems allowing for the possibility that these parasite and host proteins are likely to take on a range of conformations in vitro and in vivo. In combination with the P. vivax invasion-inhibition assay, it is possible to evaluate consistency between binding assays and interference with P. vivax infection of human erythrocytes. Overall, the approach we have taken demonstrates that these in vitro strategies may identify meaningful correlates of naturally induced immunity to P. vivax.

From the binding-inhibition assays, which allow PvDBP or the Duffy antigen to assume multiple conformations, we observed that PvDBP–Duffy binding occurred as expected in the absence of anti-PvDBPII–specific antibody. This interaction was inhibited by addition of the anti-PvDBPII–specific antibodies in a dose-dependent fashion in all three of the binding-inhibition assays. Importantly, this was true even for antibodies affinity purified from people exposed to natural infections. Binding inhibition of approximately 50% was observed to occur at dilutions of the rabbit anti-PvDBPII serum between 1:1,000 and 1:3,200. Comparisons between the nDARC-Ig ELISA and erythrocyte-binding assay were similar, and inhibition of rosette formation in the COS7 cell assay system occurred at a lower antibody concentration. Using the human PvDBPII-specific antibody, binding inhibition was observed between 3 and 75 μg/ml. These inhibitory antibody concentrations are consistent with those observed in studies investigating P. falciparum MSP1_19_ (merozoite surface protein-1 19) binding [44].

Of further technical interest, it is important to note that prior to affinity purification of anti-PvDBPII Ab, the pooled human sera showed a 2-fold higher ELISA reactivity to PvMSP1_19_ when compared with PvDBPII. This potentially resulted from the ubiquitous expression of PvMSP1_19_ on the parasite surface and from high levels of P. vivax protein found in the blood of P. vivax–infected patients. However, after affinity purification of anti-PvDBPII Ab from the pooled plasma, reactivity specific for PvDBPII was 4-fold higher than that of PvMSP1_19_ by ELISA (see Figure S2). We cannot completely exclude the possibility that high-avidity antibodies directed against PvMSP1_19_, or against other P. vivax merozoite surface proteins, were in the enriched anti-PvDBPII Ab preparation and contributed to inhibition of P. vivax invasion. One laboratory-adapted strain of P. falciparum tested by Hodder et al. demonstrated that 20 μg/ml of anti-AMA1 antibody, isolated from pooled human sera under procedures similar to those used here, was sufficient to reduce parasite invasion of erythrocytes [45]. However, the majority of strains tested by Hodder, as well as those investigated in other previous studies of enriched P. falciparum anti-AMA1or anti-MSP1_19_ antibodies, required ≥100 μg/ml to show significant inhibition of P. falciparum invasion of human erythrocytes [44,45].

Further comparison of these in vitro binding-inhibition assays illustrates the operational advantages of the nDARC-Ig ELISA and erythrocyte-binding assay systems; these systems may promote more efficient identification of molecular strategies to block, or to induce the acquisition of relevant antibody response against P. vivax infection of human red blood cells. Although the exact structure of the nDARC-Ig molecule is not known, its does bind with high affinity to the rPvDBPII protein, to the mAb Fy6, which recognizes a linear epitope in the N-terminal region of the native Duffy antigen, and to the expected array of chemokines known to interact with the Duffy antigen [34]. The advantages of the nDARC-Ig assay include the following: it can be performed using all recombinant reagents, it is rapidly performed and is easily standardized, and it uses less than 10 μl of antiserum. The erythrocyte-binding assay evaluates the interaction between properly refolded rPvDBPII and the native Duffy antigen receptor in human red blood cells. The protein–protein interactions in this assay may therefore better mimic the parasite–host system, and antibody inhibition may show close comparability to vaccine-induced immune response. Analysis of binding inhibition performed by flow cytometry is quantitative, rapid, and utilizes, at most, 20 μl of antiserum. A limitation of this assay includes the possibility that individual serum samples may contain antibodies that are cross-reactive to erythrocytes provided by different donors for the assay, resulting in erythrocyte agglutination. Relative antigen–red cell binding could also vary between assay donors depending on a range of factors influencing red blood cell structure and function. An advantage of the COS7 cell assay is that it avoids expression and purification of recombinant antigen.

Although the rabbit and human anti-PvDBPII Ab demonstrated between 75 and 100% inhibition of binding in the three in vitro assay systems described above, similar concentrations of antibodies inhibited merozoite invasion by no more than 50–60%. This difference was not surprising since the antibodies were pre-incubated with rPvDBPII or COS7 cells expressing PvDBPII in the binding-inhibition assays. By contrast, the merozoite only expresses PvDBP on the surface of its apical end just before rupturing the erythrocyte [18]. Therefore, the antigen may not be readily available for binding to the anti-PvDBPII Ab and higher concentrations may be required to inhibit parasite invasion. This observation raises a number of considerations that may influence immune recognition and response against PvDBPII. For example, the same limited exposure of the host immune system to PvDBP may contribute to the failure by some residents of P. vivax–endemic areas to develop humoral immunity to PvDBPII [33]. As implied by our previous findings, the highly polymorphic nature of PvDBP may also confound development of antibodies capable of inhibiting merozoite invasion of red blood cells [41]. In this study, we prepared rabbit and human anti-PvDBPII Ab using Sal 1 rPvDBPII. Since the parasite invasion experiments were performed with wild strains of *P. vivax,* there may be sufficient differences in antibody recognition of critical binding epitopes leading to reduced efficacy of invasion inhibition. Ultimately, we can examine this more closely by comparing antibodies raised against variant PvDBP alleles.

The low number of parasites available from donor blood samples contributed to limitations in the number of conditions that could be evaluated in the in vitro invasion-inhibition assays. At the present time, donor parasitemia and the P. vivax preference to invade reticulocytes [46–49] introduce significant limitations to the types of studies that can be performed. The lengthy transport time of the blood sample from the clinic to the research laboratories and the procedures used to remove white blood cells from the samples resulted in a further reduction in parasitemia. Recent developments in P. vivax culturing techniques may reduce the attrition of parasitized cells observed in culture preparation. Additionally, by supplementing P. vivax cultures with enriched reticulocyte preparations [28], it may be possible to improve observation of P. vivax erythrocyte-invasion events in vitro. Finally, by adapting flow-cytometric methods used to measure P. falciparum growth and development for use with *P. vivax,* it may be possible to improve sensitivity of the parasite invasion assays for samples with low parasitemias [50].

In conclusion, our study provides evidence that antibodies against PvDBPII inhibit binding to the Duffy receptor and interfere with P. vivax invasion of human red blood cells. As our recent study reported that reduced erythrocyte Duffy expression by Duffy-negative heterozygotes lowers susceptibility to P. vivax blood-stage infection [11,12], our combined results suggest that there may be an important threshold of PvDBP–Duffy interaction necessary for parasite invasion of the human red blood cell. This emphasizes that PvDBP is a critical parasite invasion ligand to target in P. vivax vaccine development efforts. Therefore, as the binding-inhibition assays employed here allow quantitative assessments of the molecular partners involved in P. vivax human red cell invasion, it becomes possible to perform highly relevant assays in high-throughput format to evaluate functional correlates of immunity against P. vivax blood-stage infection and disease in population-based studies.

## Supporting Information

Figure S1 Evaluation of rPvDBPII Protein Expression and Refolding (A) Coomassie-stained SDS-PAGE gel, showing M, protein size markers, rPvDBPII, refolded protein, and rPvDBPII+DTT, denatured protein after treatment with 10 mM dithiothreitol.
(B) Results of an erythrocyte-binding assay, with refolded rPvDBPII after preadsorption with Duffy-positive and Duffy-negative erythrocytes. The binding assay was performed by incubating erythrocytes with refolded protein, and the reaction mixture was then layered over dibutylpthalate (Sigma) and centrifuged to collect erythrocytes. Bound protein was eluted from erythrocytes with 300 mM NaCl; unbound protein demonstrates that there was the same amount of protein added to each well. The rPvDBPII protein was detected by Western blotting with anti-HIS monoclonal antibodies conjugated to horseradish peroxidase. Note that the refolded protein forms two bands that might represent slight variations in the way the protein refolds. This same pattern has been observed previously by Singh et al. [25].
(30 KB PDF)

Figure S2 ELISA to Determine Titer of Antibodies Rabbit antiserum was raised against the rPvDBPII Sal 1 variant. (A) ELISA titers of the rabbit antiserum to rPvDBPII Sal 1 and C variants. (B) Titers of affinity-purified human anti-PvDBPII Ab when attached to Sal 1 and C variants of rPvDBPII and to recombinant PvMSP1_19_, a highly immunogenic antigen widely recognized by human anti-P. vivax antibodies [51].
(22 KB PDF)

Figure S3 COS Cell Binding Assay The COS cell binding assay reports the average of three independent experiments, each performed in triplicate on the rabbit anti-PvDBPII serum. The inoculated rabbit serum blocked COS7 cells expressing PvDBPII from forming rosettes in a dose-dependent fashion, leading to an indication that the PvDBPII was correctly folded and that antibodies directed against this protein were effective in interrupting the PvDBPII–Duffy interaction.
(14 KB PDF)

## Abbreviations

Anti-PvDBPII Ab: antibodies against region two of the Plasmodium vivax Duffy binding protein
anti-PvDBPII serum: serum containing antibodies against region two of the Plasmodium vivax Duffy binding protein
DARC: Duffy antigen/receptor for chemokines
DBP: Duffy binding protein
MSP1_19_: merozoite surface protein-1 19
nDARC-Ig: first 60 codons of human Duffy antigen receptor for chemokines ligated to Fc region of human IgG
PvDBP: Plasmodium vivax Duffy binding protein
PvDBPII: region two of the Plasmodium vivax Duffy binding protein
PvMSP1_19_: Plasmodium vivax merozoite surface protein-1 19
rPvDBPII: recombinant region two of the Plasmodium vivax Duffy binding protein
Sal 1 and C: Salvador 1 and C recombinant P. vivax DBPII variants
