# Supplementary material for: Plasmodium vivax Invasion of Human Erythrocytes Inhibited by Antibodies Directed against the Duffy Binding Protein
Source: PLoS Med. 2007 Dec 18;4(12):e337. doi: 10.1371/journal.pmed.0040337 (PMC2140086; doi:10.1371/journal.pmed.0040337)
Supplement: Supplementary file 1 [file pmed.0040337.sg001.pdf]

Supporting Information #1

Evaluation of rPvDBPII protein expression and refolding. These studies were initiated by expression of recombinant PvDBP region two (rPvDBPII) containing the minimal binding region for *P. vivax* interaction with the Duffy antigen. The Sal 1 and PNG "C" variants of PvDBPII (aa 251 to 566) with a 6-His tag and stop codon were cloned into the Gateway pDONR-vector (Invitrogen Corp., Carlsbad, CA) using a TA cloning strategy. The construct was then cloned into Gateway pET-DESN^{TM} 42 vector (Invitrogen Corp., Carlsbad, CA.) and expressed in codon optimized BL21 *E. coli* (Invitrogen Corp., Carlsbad, CA). The cell pellet was lysed twice using CelLytic B Bacterial Cell Lysis Reagent (Sigma, St Louis, MO) with deoxyribonuclease I and protease inhibitors in order to release the insoluble protein product. In order to harvest sufficient quantities of the recombinant protein, it was necessary to first denature the highly insoluble protein in 8M guanidine hydrochloride. Each gram of insoluble protein was mixed with 50 mL of fresh solubilization buffer (10 mM Tris, pH 8.0, containing 8 M guanidine hydrochloride, 10 mM DTT). The protein was then purified on a NiNTA column (Hi(s)-Trap Column; Amersham Biosciences, Piscataway, NJ) using wash buffer (PBS, pH 7.6-7.9, 6 M Guanidine HL, 10 mM imidazole) and elution buffer (PBS pH 7.6-7.9, 6 M Guanidine HL, 250 mM imidazole). After purification on a nickel nitrilotriacetic acid column, the protein was refolded by 100-fold rapid dilution in an arginine-based buffer and further purified using ion-exchange chromatography and gel filtration. To induce proper refolding of rPvDBPII one volume of the purified protein was immediately placed into 100 volumes of fresh refolding buffer (50 mM NaH_2PO_4, 1 mM reduced glutathione, 0.1 mM oxidized glutathione, 1 M urea, and 0.5 M arginine pH 7.2) and stirred for at least 36 hr at 4°C. The refolded protein was then dialyzed (10,000 MW cut-off) in 50 mM NaH_2PO_4, and 1 M urea pH 6.5 for 12 hr with three exchanges of buffer and further purified by ion exchange column chromatography. The refolded protein was eluted from

the ion exchange column by NaCl gradient (0.2 M to 1 M); protein elution maximum occurred at 0.8 M. The now soluble protein was rinsed in a dialyzing buffer (0.05 M sodium phosphate buffer and 0.15 M NaCl, pH 7.2). The protein was further purified by FPLC on a HiLoad 16/6- Superdex 75 prep grade column (Amersham Biosciences, Piscataway, NJ). Evidence for refolding of the purified rPvDBPII protein is shown in Figure S1A illustrating that the refolded protein exhibits a faster mobility than denatured (DTT-treated) protein. This mobility shift suggests that DTT treatment reduces expected di-sulfide bonds in the refolded protein. Results in Figure S1B demonstrate that the refolded protein binds to erythrocytes of Duffy-positive but not Duffy-negative individuals (Figure S1B left-hand portion); unbound rPvDBPII (Figure S1B right-hand portion) shows that equal amounts of the protein were added to erythrocytes from both donors.

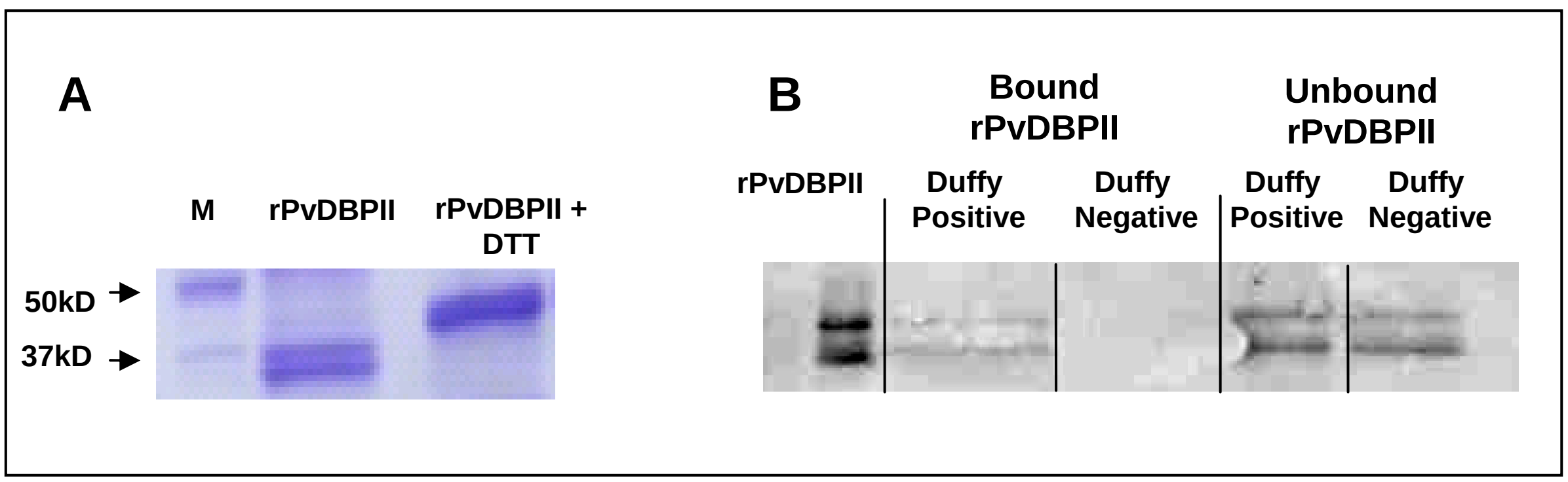

Figure S1. Evaluation of rPvDBPII protein expression and refolding.

Coomassie stained SDS-PAGE gel (A) showing M = Protein size markers, rPvDBPII = refolded protein, and rPvDBPII+DTT = denatured protein after treatment with 10 mM dithiothreitol (DTT). Results of an erythrocyte binding assay (B) with refolded rPvDBPII after preadsorption with Duffy-positive and Duffy-negative erythrocytes. The binding assay was performed by incubating erythrocytes with refolded protein, the reaction

mixture was then layered over dibutylpthalate (Sigma) and centrifuged to collect erythrocytes. Bound protein was eluted from erythrocytes with 300 mM NaCl, unbound shows that there was the same amount of protein added to each well. The rPvDBPII protien was detected by Western blotting with anti-HIS monoclonal Ab conjugated to HRP. Note that the refolded protein forms 2 bands that might represent slight variations in the way the protein refolds. This same pattern has been observed previously by Singh et al. [1].

Reference

1. Singh S, Pandey K, Chattopadhayay R, Yazdani SS, Lynn A, et al. (2001) Biochemical, biophysical, and functional characterization of bacterially expressed and refolded receptor binding domain of Plasmodium vivax duffy-binding protein. J Biol Chem 276: 17111-17116.
