# Supplementary material for: Plasmodium vivax Invasion of Human Erythrocytes Inhibited by Antibodies Directed against the Duffy Binding Protein
Source: PLoS Med. 2007 Dec 18;4(12):e337. doi: 10.1371/journal.pmed.0040337 (PMC2140086; doi:10.1371/journal.pmed.0040337)
Supplement: Supplementary file 2 [file pmed.0040337.sg002.pdf]

Supporting Information #2

Antibody Titer by ELISA

As the *P. vivax* Duffy Binding Protein (PvDBP) is highly polymorphic [1] we over-expressed and further purified both Sal 1 and the C variants following methods previously developed by Singh et al. [2] (Figure S1). These alleles differ at 7 amino acid residues in the region hypothesized to engage in binding to the Duffy receptor. Using region two of a recombinant PvDBP protein (rPvDBPII), Sal 1 variant, we developed a rabbit anti-PvDBPII serum that recognized the recombinant protein with an endpoint titer of approximately 1:500,000. We then addressed the influence of PvDBP polymorphism by comparing the rabbit antiserum recognition between the Sal 1 and C variants.

To determine the titer of both rabbit and human Ab, rPvDBPII variants Sal 1, C , or $PvMSP1_{19}$ (1 µg /ml) were resuspended in 50 µl of coating buffer ($NaHCO_3$; pH 9.6) and added to Immulon 4 ELISA plates (Promega, Madison, WI) and incubated overnight at 4°C. The plates were blocked with 5% milk and washed twice in PBS; 50 µl of test plasma or serum was added at the designated dilutions to individual wells and incubated overnight at 4°C. After washing twice in PBS the samples were incubated with 1:2000 dilution of alkaline-phosphate conjugated goat-anti-human (or anti-rabbit) IgG (Jackson ImmunoResearch, West Grove, PA), incubated for 2 hr at 37°C and washed twice in PBS. The substrate, p-nitrophenyl phosphate, was then added and $OD_{422\ nm}$ measured using a Molecular Devices VersaMax™ microtiter plate reader (Sunnyvale, CA).

Results in Figure S2A show that there were no significant differences in binding of the rabbit anti-PvDBPII serum to either PvDBPII variant. Using the rPvDBPII Sal 1 variant we affinity purified human anti-PvDBPII Ab from the sera of 14 Papua New Guineans who displayed high antibody titers to rPvDBPII. We found no evidence of a difference in the human anti-PvDBPII Ab recognition of recombinant proteins variants C and Sal 1

(Figure S2B). To evaluate the relative enrichment and specificity of anti-PvDBPII Ab, following affinity column purification, we tested the ability of the antibody to recognize another *P. vivax* merozoite surface protein, $PvMSP1_{19}$. Results in Figure S2B suggest that a small level of anti-$PvMSP1_{19}$ was measured; however the predominant Ab activity was directed against PvDBPII. Of note, the pooled human sera before affinity purification showed a higher ELISA reactivity to $PvMSP1_{19}$ (O.D. 1.7) compared with PvDBPII (O.D. 0.9). This resulted potentially from the ubiquitous expression of $PvMSP1_{19}$ on the parasite surface and from high levels of *P. vivax* protein found in the blood of *P. vivax* infected patients.

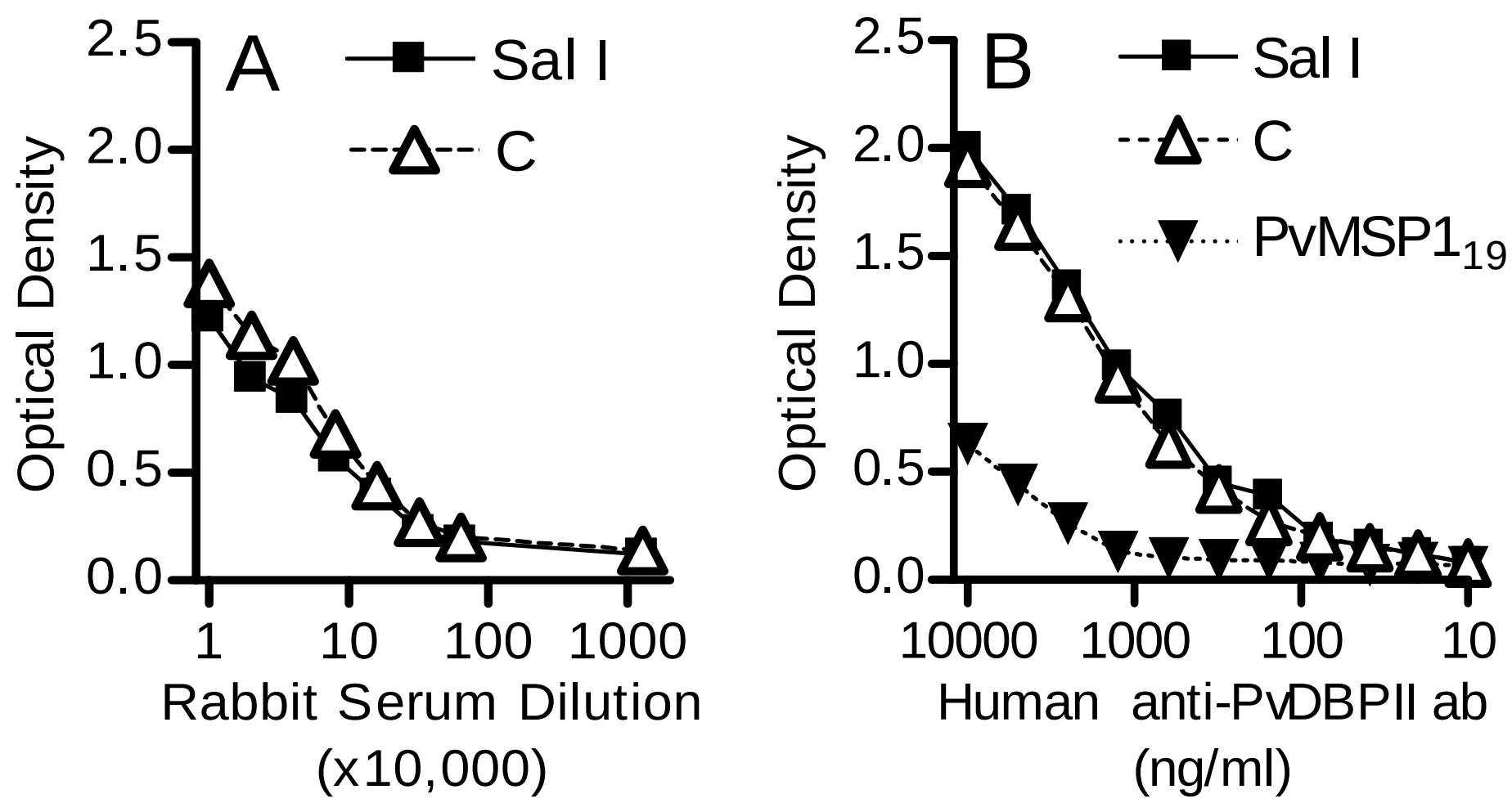

Figure S2. ELISA to Determine Titer of Antibodies.

Rabbit antiserum was raised against the rPvDBPII Sal 1 variant. Panel A shows the ELISA titers of the rabbit antiserum to rPvDBPII Sal I and C variants. Panel B shows the titers of affinity purified human anti-PvDBPII Ab when attached to Sal I and C variants of rPvDBPII and to recombinant $PvMSP1_{19}$, a highly immunogenic antigen widely recognized by human anti-*P. vivax* Ab [3].

References

1. Cole-Tobian JL, Biasor M, King CL (2005) High complexity of Plasmodium vivax infections in Papua New Guinean children. Am J Trop Med Hyg 73: 626-633.

2. Singh S, Pandey K, Chattopadhayay R, Yazdani SS, Lynn A, et al. (2001) Biochemical, biophysical, and functional characterization of bacterially expressed and refolded receptor binding domain of Plasmodium vivax duffy-binding protein. J Biol Chem 276: 17111-17116.

3. Soares IS, Rodrigues MM (2002) Immunogenic properties of the Plasmodium vivax vaccine candidate MSP1(19) expressed as a secreted non-glycosylated polypeptide from Pichia pastoris. Parasitology 124: 237-246.
