# Supplementary material for: Plasmodium vivax Invasion of Human Erythrocytes Inhibited by Antibodies Directed against the Duffy Binding Protein
Source: PLoS Med. 2007 Dec 18;4(12):e337. doi: 10.1371/journal.pmed.0040337 (PMC2140086; doi:10.1371/journal.pmed.0040337)
Supplement: Supplementary file 3 [file pmed.0040337.sg003.pdf]

Supporting Information #3

COS7 Cell-based Binding Inhibition Assay.

COS7 cells were transiently transfected with plasmid expressing GFP and HSVgD1 and PvDBPII (Sal 1 variant) as previously described (1). Here binding inhibition studies were performed by first pre-incubating COS7 cells expressing the PvDBPII protein with rabbit anti-PvDBPII serum at 1:100 - 1:6400 dilutions (1 hr at 37°C, 5% CO_2 in Dulbecco's modified Eagle's media (DMEM)). To this mixture, Duffy-positive human erythrocytes were added and further incubated (2 hr at room temperature) to allow COS7 cell:human erythrocyte rosette formation. Peripheral venous blood from Duffy-positive and -negative donors was collected in 8.5 ml ACD solution A vacutainers, centrifuged at 400g for 3 min, plasma was removed, and pelleted erythrocytes were washed three times with 10 ml of DMEM. The Duffy antigen status was determined as previously described (2). Relative binding in the presence of rabbit anti-PvDBPII serum compared with pre-bleed serum was determined by counting rosettes observed in 30 fields of view (200x). Results in Figure S3 demonstrate that pre-incubation of COS7 PvDBPII transfectants with a 1:3200 dilution of the rabbit anti-PvDBPII serum inhibited binding to erythrocytes from Duffy-positive donors by >50% compared with the pre-bleed serum; rosette disruption correlated with increasing concentration of the rabbit PvDBPII serum. The COS7 cell binding assay using the affinity-purified human anti-PvDBPII Ab was not used because of the limited amount of Ab available.

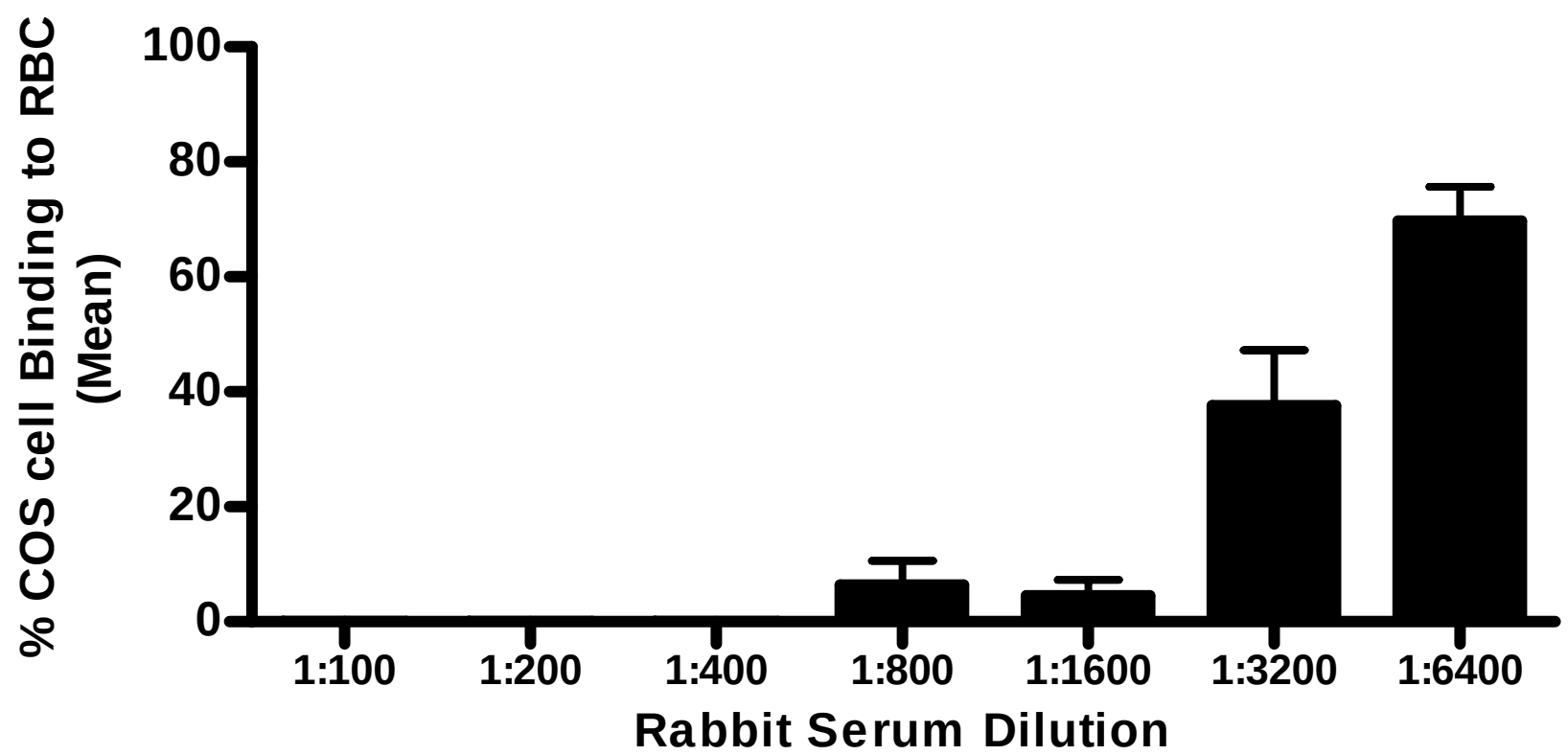

Figure S3. COS Cell Binding Assay

The COS cell binding assay reports the average of three independent experiments, each performed in triplicate on the rabbit anti-PvDBPII serum. The inoculated rabbit serum blocked COS7 cells expressing PvDBPII from forming rosettes in a dose dependant fashion leading to an indication that the PvDBPII was correctly folded and that Ab directed against this protein were effective in interrupting the PvDBPII:Duffy interaction.

References

1. VanBuskirk KM, Cole-Tobian JL, Baisor M, Sevova ES, Bockarie M, King CL , Adams JH (2004) *J Infect Dis* 190:1556-1562.

2. Tournamille C, Le Van Kim C, Gane P, Le Pennec PY, Roubinet F, Babinet J, Cartron JP , Colin Y (1998) *Blood* 92:2147-2156.
